# Epidemiological statistics of congenital thumb duplication in the Chinese population

**DOI:** 10.1186/s13018-021-02567-3

**Published:** 2021-08-09

**Authors:** Yingling Yao, Haolin Zhou, Lianyong Li, Guoxin Nan

**Affiliations:** 1 Ministry of Education Key Laboratory of Child Development and Disorders, National Clinical Research Center for Child Health and Disorders (Chongqing), China International Science and Technology Cooperation Base of Child Development and Critical Disorders, Stem Cell Biology and Therapy Laboratory, Chongqing Key Laboratory of Pediatrics, Children’s Hospital of Chongqing Medical University, Chongqing, 400014 China; 2 Department of Orthopaedics, Children’s Hospital of Chongqing Medical University, Chongqing, China; 3 Department of Pediatric Orthopedics, Shengjing Hospital of China Medical University, No.36 Sanhao Street, Heping District, Shenyang City, 110004 China

**Keywords:** Thumb polydactyly, Southwest China, Northeast China, Thumb duplication, Wassel classification

## Abstract

**Background:**

Thumb duplication is a very common congenital malformation. This study describes and compares the phenotypic manifestations of polydactyly between southwest and northeast China. However, previous studies had a limited sample size. Therefore, this study used a large sample.

**Methods:**

A total of 3549 well-characterized thumb duplication cases were divided into group A (southwest China) and group B (northeast China).

**Results:**

In group A and group B, the left-to-right ratio was 1:1.5 and 1:1.75, respectively, and the female-to-male ratio was 1:1.5 and 1:1.58, respectively.

**Conclusions:**

There were no significant differences in gender distribution or the distribution of left and right polydactyly between the two groups, but the distribution of bilateral polydactyly was significantly different.

## Introduction

Thumb duplication is a very common congenital malformation. Preaxial polydactyly is the most common duplication in Caucasian and Asian populations, and it occurs in 0.8–1.4 cases per 1000 births [1]. Abnormal expression of morphogens, such as Hox genes, bone morphogenic proteins, LMBR1, Gli-3, and increased duplication of ZRS region has been associated with thumb duplication [2–5].The Wassel system, which was developed in 1969, has become the universal classification system for thumb duplication due to its simplicity [1]. Type IV thumb duplication is the most common type, and it is followed by type II and then type VII [5–7].

Several series have reported the distribution of different types of thumb polydactyly in domestic and foreign populations. However, there is no large sample study of polydactyly in northeast and southwest China. There are differences in climate, topography, ethnicity, economic level, diet, and medical level in northeast and southwest China. The northeast is mainly a plain with a cold climate, while the southwest is dominated by a mountainous plateau basin with a subtropical monsoon climate and a large number of ethnic minorities. Therefore, the purpose of this study is to describe the epidemiological characteristics of thumb duplication based on a statistical analysis of the Chinese population and to elucidate whether the clinical and epidemiological characteristics of thumb duplication in southwest China differ from its clinical and epidemiological characteristics in northeast China.

## Methods

Patients with thumb duplication were identified between 2012 and 2019 at Children’s Hospital of Chongqing Medical University (southwest China) and China Medical University (northeast China). The diagnosis of thumb duplication is done by two or more clinicians. The thumb duplication classification is established by two doctors after independent judgment; If there is a disagreement, the typing result is discussed together after a third doctor has made a judgment. The classified according to the Wassel classification (Table 1).

**Table 1:** Classification of thumb polydactyly used in the current study

Wassel I	Bifid distal phalanx
Wassel II	Duplicated distal phalanx
Wassel III	Bifid proximal phalanx
Wassel IV	Duplicated proximal phalanx
Wassel V	Bifid metacarpal
Wassel VI	Duplicated metacarpal
Wassel VII	Thumb duplication with triphalangism
Rudimentary	Small appendage with a narrow pedicle

A total of 3549 cases from different families with thumb duplication were included in this study.

The group with thumb duplication recruited from the hospital in southwestern China was designated as group A, and the group with thumb duplication recruited from the hospital in northeastern China was designated as group B. Compare the gender and left-right differences of thumb duplication patients in the two regions.

Statistical analysis was performed using SPSS 23.0 software, qualitative data were described by percentages; count data were expressed as rates or composition ratios, and differences between groups were analyzed using the χ^2^ test. *P*<0.05 (two-sided).

This study has been approved by the Institutional Review Board (IRB) of our hospital.

## Results

In group A, there were 2463 cases of thumb duplication. Of these, 333 were cases of bilateral thumb duplication. A positive family history was recorded in 160 cases. In total, there were 1492 males, who accounted for 60.6% of the population, and 971 females, who accounted for 39.4% of the population. The male-to-female ratio was 1.5:1, and the left-to-right thumb duplication ratio was 1:1.5 (1121 with left thumb duplication and 1675 with right thumb duplication). In case of unilateral thumb duplication, polydactyly was observed in 1274 males and 857 females (Table 2).

**Table 2:** Distribution of thumb polydactyly according to gender (group A)

Sex	n	Unilateral	Bilateral
Male	1492	1274 (85.4)	218 (14.6)
Female	971	857 (88.3)	114 (11.7)
*χ2*		4.157	
*P*		0.041	

The exclusion criteria of classification are as follows: [1] re-admission to the hospital for lesions and deformities, [2] imaging done outside the hospital without imaging data from our hospital. The types of polydactyly observed in the current study cohort are shown in Table 3. There were 2463 cases of thumb duplication, out of which 200 (8.0%) were excluded and 2263 (2562 fingers) were included. Among the included cases, 238 (11%) did not fit the classic Wassel types, and 5 cases did not fit into any of the Rotterdam classification types. Type IV thumb duplication accounted for the overwhelming majority of the patients (35.3%, 905/2562). Further, type II thumb duplication was observed in 13.7% (351/2562) of the cases, type V in 9.5% (243/2562) of the cases, and type VII and type I in 9.1% (233/2562) and 2.6% (67/2562) of the cases, respectively (Table 3). So, the total of 2025 cases (2279 fingers) of thumb duplication has been classified by Wassel. The present findings are compared with those from other studies at home and in other countries in Table 4. Further, as shown in Table 5, there was no significant difference in the distribution of the different types with regard to sex (χ^2^ = 12.146, *P* = 0.096) or affected side (χ^2^ = 11.134, *P* = 0.133). Bilateral rudimentary thumb duplication was more common than unilateral rudimentary thumb duplication (*P* < 0.05, Table 5).

**Table 3:** Distribution of the types of thumb polydactyly in 2562 hands (group A)

Wassel type	Male	Female	Left	Right	Unilateral	Bilateral	Total	(%)
(Number of hands)								
**I**	41	26	18	49	52	15	67	2.6
**II**	194	157	138	213	295	56	351	13.7
**III**	127	89	91	125	178	38	216	8.4
**IV**	580	325	348	557	731	174	905	35.3
**V**	149	94	90	153	177	66	243	9.5
**VI**	62	29	42	49	63	28	91	3.6
**VII**	136	97	91	142	180	53	233	9.1
**Rudimentary**	111	62	80	93	95	78	173	6.8
**Others**	168	115	113	170	193	90	283	11.0
**Total**	1568	994	1011	1551	1964	598	2562	100

**Table 4:** Comparison of the proportions of Wassel types across several domestic and international studies

Wassel type	Our data		China, Su et al.		The Middle East, Al-Qattan et al.		Japan, Islam et al.		The UK, Naasan A et al.	
	Number	%	Number	%	Number	%	Number	%	Number	%
I	67	2.9	5	1.7	1	0.5	6	3.8	2	4.7
II	351	15.4	43	14.5	21	9.5	36	22.8	13	30.2
III	216	9.5	16	5.4	31	14.1	8	5.1	8	18.6
IV	905	39.7	88	29.6	77	35	53	33.6	9	20.9
V	243	10.7	23	7.8	13	5.9	10	6.3	4	9.3
VI	91	4.0	20	6.7	31	14.1	11	6.9	4	9.3
VII	233	10.2	83	27.9	28	12.7	11	6.9	3	7.0
Rudimentary	173	7.6	19	6.4	18	8.2	23	14.6		
Total	2279	100%	297	100%	220	100%	158	100%	43	100%

**Table 5:** Phenotypic characteristics of thumb duplication in group A

Variables	Total	I		II		III		IV		V		VI		VII		Rudimentary		*P*
	N	N	%	N	%	N	%	N	%	N	%	N	%	N	%	N	%	
Sex																		
Male	1400	41	2.9	194	13.9	127	9.1	580	41.4	149	10.6	62	4.4	136	7.9	111	7.9	**0.096**
Female	879	26	3.0	157	17.9	89	10.1	325	37.0	94	10.7	29	3.3	97	11.0	62	7.1	
Laterality																		
Unilateral	1771	52	2.9	295	16.7	178	10.1	731	41.3	177	10.0	63	3.6	180	10.2	95	5.4^a^	**<0.001**^**b**^
Bilateral	508	15	3.0	65	11.0	38	7.5	174	34.3	66	13.0	28	5.5	53	10.4	78	15.4^a^	
Sidedness																		
Left	898	18	2.0	138	15.4	91	10.1	348	38.8	90	10.0	42	4.7	91	10.1	80	8.9	**0.133**
Right	1381	49	3.5	213	15.4	125	9.1	557	40.3	153	11.1	49	3.5	142	10.3	93	6.7	

^a^Significant for the rudimentary group compared with other Wassel types

^b^Significant difference between groups

In group B, out of 1086 patients, 665 were male and 421 were female. Of these, 89 had bilateral thumb polydactyly. The male-to-female ratio was 1.58:1, and the left-to-right polydactyly ratio was 1:1.75 (362 with thumb polydactyly on the left hand and 635 with thumb polydactyly on the right).

There were no significant differences between groups A and B in terms of either gender distribution or left-right sides (*P* > 0.05).

The distribution of bilateral polydactyly was significantly different between the two groups (χ^2^ = 20.395, *P* < 0.001, Table 6).

**Table 6:** Phenotypic characteristics of thumb duplication between group A and group B

Variables	Total		Aa		Bb		χ^2^	P
	Number	%	Number	%	Number	%		
Sex								
Male	2157	60.8	665	61.2	1492	60.6	0.137	0.712
Female	1392	39.2	421	38.8	971	39.4		
Laterality								
Left	1150	32.4	362	33.3	788	32.0	0.618	0.432
Right	1977	55.7	635	58.5	1342	54.5	4.851	0.028
Bilateral	422	11.9	89	8.2	333	13.5	20.395	<0.001c

^a^Thumb duplication features of the group A

^b^Thumb duplication features of the group B

^c^Significant for each group compared with their controls

## Discussion

This study analyzed and compared the demographic and clinical characteristics of thumb duplication between southwestern and northeastern China. The obtained findings and the results obtained from the large sample size of this study could be a valuable resource for comparing the features of thumb duplication in the Chinese population with those in other populations, and could promote research on the phenotypic variability of this condition and factors related to it.

This study showed that there were no significant differences between northeast and southwest China in terms of either gender distribution or left-right sides, but the distribution of bilateral polydactyly was significantly different. However, there are differences between the research data of other countries and the research results of this paper. For example, Ozalp et al. in Turkey and Islam et al. in Japan reported a male-to-female ratio of 1:1, and the results reported by Su et al. in China and Naasan et al. in Hong Kong both showed that the male-to-female ratio was 2:1 and the left-to-right thumb polydactyly ratio was 1:1.5 [8–11]. The difference in the ratios indicates that the incidence rates in other countries in Europe and Asia are different from those in China. This difference may be attributable to differences in race, environment, economic level, social medical security, diet, and national health awareness. Additionally, the differences between Turkey, Japan, and China are mainly attributed to differences in ethnicity. However, as this dataset is also different from other domestic datasets, it is possible that other factors, such as environment, diet, geography, and sample size, are responsible for the difference in incidence.

In all races, Wassel type IV seems to be the most common, while Wassel type I seems to be the least common thumb polydactyly. However, there are differences in the specific proportions of each type reported. The results of this study in southwestern China show that type IV accounts for 39.7% of the affected population, while the proportion reported in another domestic study by Su et al., the Middle East study by Al-Qattan et al., the Japan study by Islam et al., and the UK study by Naasan et al. show that type IV accounts for 29.6%, 35%, 33.6%, and 20.9% of the respective populations [10–12].This is different from our statistical results and may be associated with the sample size. This group of data has a large sample size, similar to the previous reports at home and abroad. At present, the classification is mainly based on imaging. However, for infants and young children, it is often difficult to distinguish the epiphysis from the ossification center by radiography, and this affects the classification of type IV, V, and VII thumb polydactyly. In the case data from this group, difficulty in classification before surgery was usually solved by intraoperative classification.

Although the Wassel system represents a universal system of classification of thumb duplication because of its simplicity, it does not represent all types of thumb duplication [13]. Therefore, others have attempted to improve upon the Wassel system, including Buck-Gramcko, Upton, and Flatt, with the Rotterdam classification [1].A study by Dijkman et al. compared the reliability of the Wassel and Rotterdam classifications [7]. Out of a study population of 520 cases, only 60% could be classified using the Wassel classification, compared with 100% using the Rotterdam classification. However, Su et al. showed that only 8.6% of hands could not be classified with the Wassel classification system [11]. In comparison, in the present study, a total of 238 fingers (11%) did not fit into the classic Wassel types. A study by Hu et al. also showed that adding a hypoplastic subtype to the Wassel-Flatt can classify most of previously unclassifiable thumbs [14]. Therefore, it is necessary to propose a new classification method to supplement the existing Wassel classification.

In this regard, Gao et al. report a new classification that can be used to comprehensively describe the clinical features of the terminal phalanx in congenital thumb duplication and the surgical procedure that can be adopted for each type with satisfactory results [15].Additionally, Chung et al. developed a new classification method that is more correlated with the therapeutic approach [16]. Moreover, He et al. proposed atypical Wassel type VI and formulated corresponding treatment plans, with satisfactory treatment results and reduced complications [17]. For special cases that cannot be classified into the Wassel classification system, it is necessary to increase the sample size, to summarize the pathological and anatomical characteristics, and to further classify them to overcome the shortcomings of the existing Wassel typing methods and guide clinical treatment.

To conclude, this study could enhance our understanding of the distribution of thumb duplication types based on sex, affected side, and genetic inheritance in the Chinese population. Additionally, the findings are valuable in terms of exploring the prevalence of polydactyly-associated congenital anomalies in the Chinese population by means of epidemiological information on thumb duplication. However, this study only has case data from Southwest China and Northeast China and does not include cases across the country. If conditions permit, we will conduct a multi-center study to collect more cases.

## Data Availability

The datasets used and/or analyzed during the current study are available from the corresponding author on reasonable request.
